# Short-Term Outcome of Combined Anterior Cruciate Ligament Reconstruction and Anterolateral Ligament Reconstruction with Suture Tape Augmentation

**DOI:** 10.3390/jcm14238283

**Published:** 2025-11-21

**Authors:** Chieh-An Chuang, You-Hung Cheng, Cheng-Pang Yang, Chen-Heng Hsu, Chung-Yu Chen, Yi-Hsuan Lin, Huan Sheu

**Affiliations:** 1 Department of Orthopaedic Surgery, Keelung Chang Gung Memorial Hospital, Keelung 204, Taiwan; b9702055@cgmh.org.tw; 2 Department of Orthopaedic Surgery, Bone and Joint Research Center, Linkou Chang Gung Memorial Hospital, Taoyuan 333423, Taiwan; 3 Comprehensive Sports Medicine Center, Linkou Chang Gung Memorial Hospital, Taoyuan 333423, Taiwan; 4 Department of Orthopaedic Surgery, Linkou Chang Gung Memorial Hospital, Taoyuan 333423, Taiwan

**Keywords:** anterior cruciate ligament reconstruction, anterolateral ligament, sutures, treatment outcome, patient reported outcome measures, internal brace

## Abstract

**Background**: Persistent rotational instability and graft failure remain major challenges following anterior cruciate ligament reconstruction (ACLR). The addition of anterolateral ligament reconstruction (ALLR) and suture tape augmentation may enhance graft protection and early stability. This study aimed to assess the short-term clinical outcomes of combined ACLR and ALLR with suture tape augmentation, hypothesizing that this technique would yield favorable results in patients with ACL injury. **Methods**: Twenty-four patients (16 males, 8 females; mean age 30.8 years) with high grade pivot shift who underwent combined ACLR and ALLR with suture tape augmentation between 2022 and 2024 were retrospectively reviewed. Objective assessments included pivot-shift grading and anterior tibial translation (ATT) using a GNRB arthrometer. Patient-reported outcome measures (PROMs) comprised the International Knee Documentation Committee (IKDC), Marx activity score, and Single Assessment Numeric Evaluation (SANE) scores. Subgroup analysis compared graft diameters < 8 mm versus ≥8 mm. **Results**: The mean follow-up period was 23.4 months. Significant improvements were observed postoperatively in IKDC (45.9→83.7), Marx (4.4→9.9), SANE (49.2→85.2), and ATT (4.7→1.1 mm) (all *p* < 0.001). Full range of motion was regained at a mean of 3.2 months. Twenty-one patients presented no pivot shift and three with grade one postoperatively. No significant differences were found between the small (<8 mm) and large (≥8 mm) graft groups. MRI at one year showed intact grafts without adverse reactions. **Conclusions**: Combined ACL and anterolateral ligament reconstruction with suture tape augmentation demonstrated promising short-term stability and functional recovery without significant complications. This technique may enhance biomechanical reinforcement of both ACL and ALL grafts and represents a viable option for patients with smaller graft diameters.

## 1. Introduction

Anterior cruciate ligament (ACL) injury is among the most common sports-related knee injuries, particularly in athletes engaged in pivoting activities [1]. Although ACL reconstruction (ACLR) remains the gold standard for restoring knee stability and function, persistent rotational laxity and graft failure continue to pose challenges [2]. Reported failure rates of 10–15% in young athletes underscore the need for ongoing refinement of surgical techniques to optimize long-term outcomes [3].

The anterolateral ligament (ALL) has been reported to be an important secondary stabilizer against anterolateral rotational instability [4,5]. Studies have shown that combined ACLR and ALL reconstruction (ALLR) improves rotational control [6,7]. Furthermore, combined ACLR and ALLR has been reported to decrease graft failure rate and revision rates of ACLR compared with isolated ACLR [8,9]. These findings have fueled increasing interest in incorporating ALLR into ACLR procedures, particularly for high-risk patients [10].

On the other hand, suture tape augmentation (STA) has emerged as a promising innovation aimed at improving knee laxity and protecting the graft during its early healing phase [11,12]. Acting as an “internal brace”, STA provides additional reinforcement to reduce micromotion and elongation under load with added graft stiffness [13,14,15]. Studies have reported encouraging outcomes for ACLR with STA, including improved stability and potentially reduced early graft failure [16].

More recently, STA has also been applied to ALL repair or reconstruction techniques in ACL-injured patients [17,18]. Different ALL reconstruction techniques have been described in the literature, including the traditional two-limb construct and the single-limb construct [19,20]. When performing combined ACLR and ALLR, the graft—often the gracilis tendon used for the ALL—may be relatively small in diameter or insufficient in length for a two-limb construct. The addition of suture tape augmentation can provide supplementary support for the single-limb gracilis graft, particularly in cases with smaller graft diameters.

As a result, while combined ACLR and ALLR have demonstrated improved control of rotational instability, the addition of suture tape augmentation in both ACL and ALL may provide further biomechanical reinforcement that protects the graft complex, particularly during the healing phase. Accordingly, the integration of ACLR, ALLR, and STA in a single procedure represents a biomechanically complementary strategy designed to enhance early stability, promote graft protection, and potentially improve short-term clinical outcomes. The aim of the study was to evaluate the short-term clinical outcomes of combined ACLR and ALLR with STA in patients presenting with ACL deficiency and pivot instability. We hypothesized that this combined strategy could provide promising early stability and favorable functional outcomes without complications.

## 2. Materials and Methods

### 2.1. Study Design and Patient Selection

This retrospective study reviewed patients who underwent combined anterior cruciate ligament reconstruction (ACLR) and anterolateral ligament reconstruction (ALLR) between 2022 and 2024. Patients were included if they presented with a high-grade pivot shift preoperatively; underwent combined ACLR and ALLR with suture tape augmentation; had at least one year of postoperative follow-up; and had complete medical records with patient-reported outcome measures (PROMs). Exclusion criteria included absence of a high-grade pivot shift, follow-up of less than one year, or incomplete clinical or PROMs data.

### 2.2. Clinical and Demographic Data Collection

Demographic and clinical information was collected, including age, sex, body mass index (BMI), time since surgery, graft diameter, tibial slope, meniscal injury, and cartilage status. Objective evaluations comprised the pivot-shift test grading, GNRB^®^ system (GNRB) for anterior tibial translation (ATT) [21], and assessment of knee range of motion (ROM). PROMs included Marx activity score, the International Knee Documentation Committee (IKDC) score, and Single Assessment Numeric Evaluation (SANE). The assessment and grading of the pivot-shift test followed previously published criteria, with grades classified as 0 (normal), 1 (glide), 2 (clunk), and 3 (locked subluxation) [22]. A high-grade pivot shift was defined as a pivot-shift test graded as 2 or 3. Return to preinjury activity level was evaluated based on both the Marx Activity Rating Scale and patient self-report. It was defined as the resumption of the same level of sports participation as before injury, with a postoperative Marx score equal to or higher than the preinjury value. For subgroup analysis, a post hoc grouping was performed. Patients were divided according to graft diameter into those with grafts smaller than 8 mm and those with grafts measuring 8 mm or greater.

### 2.3. Surgical Technique

**Figure 1:**
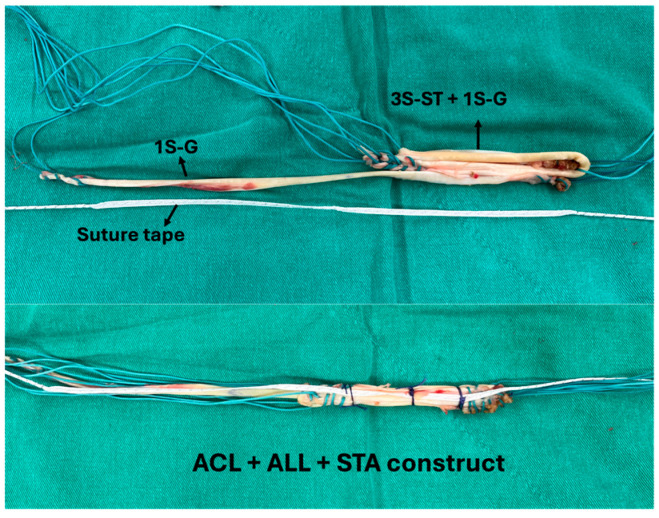
Continuous graft of combined ACL, ALL and suture tape augmentation, before and after suture integration. 3S-ST: 3-stranded semitendinosus tendon; 1S-G: 1-stranded gracilis tendon; G: Gracilis tendon; ACL: Anterior cruciate ligament; ALL: Anterolateral ligament; STA: Suture tape augmentation.

All reconstructions were performed using hamstring autografts. The graft was prepared by tripling the semitendinosus tendon and combining it with a single strand of gracilis tendon. Both tendons were sutured together to create a continuous construct, forming a four-strand ACL graft (three strands of semitendinosus and one of gracilis) and a single-strand gracilis extension for the ALL. A suture tape (FiberTape, Arthrex, Naples, FL, USA) was taken from Biocomposite Swivelock C anchor (Swivelock C; Arthrex) and was incorporated along the ACL and ALL grafts as an internal brace, as shown in Figure 1.

**Figure 2:**
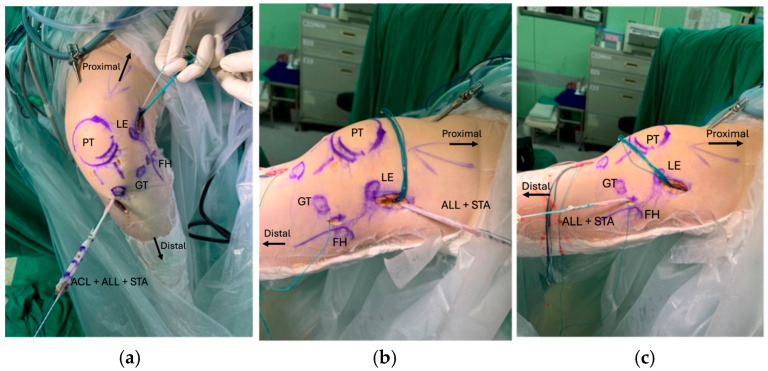
Demonstration of combined ACL and ALL and suture tape passage (**a**) Graft passage trantibial into the knee (**b**) ALL and suture tape passed out of the femur tunnel over proximal and posterior to the femoral lateral epicondyle. (**c**) ALL and suture tape passed underneath the IT band to tibial ALL point between Gerdy’s tubercle and fibula head. PT: patella; LE: lateral epicondyle; GT: Gerdy’s tubercle; FH: fibula head; ACL: Anterior cruciate ligament; ALL: Anterolateral ligament; STA: Suture tape augmentation.

**Figure 3:**
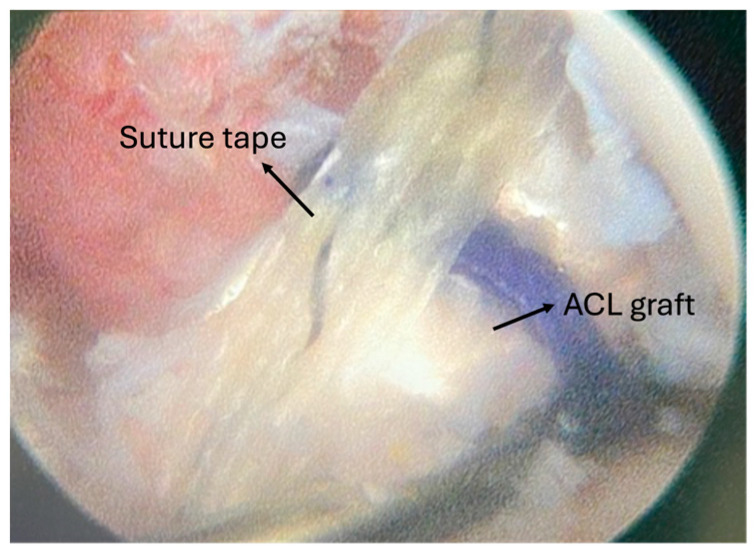
Demonstration of ACL graft and suture tape augmentation intra-articularly.

Both femoral and tibial tunnels were prepared using an outside-in technique. The femoral tunnel entry point was positioned proximal and posterior to the lateral epicondyle, with the exit directed toward the posterolateral aspect of the lateral femoral condyle at the anatomic anteromedial (AM) bundle footprint. The tibial tunnel for the ALL was located midway between Gerdy’s tubercle and the fibular head, approximately 1 cm below the joint line. After tunnel preparation, the graft was passed from tibia to femur, and the ALL portion was routed distally beneath the iliotibial band to the tibial tunnel, as illustrated in Figure 2a–c. The ALL graft was then passed into the tibial tunnel. Graft fixation was performed using bioabsorbable interference screws. The ACL graft was tensioned and fixed at 30° of knee flexion under the reverse Lachman test, with manual assistance to ensure appropriate tension. The ALL graft was fixed at full knee extension with the tibia in a neutral rotational position [23]. The suture tape was fixed alongside the grafts and finally tied together at the tibial incision site to reinforce the construct. The final intra-articular configuration is demonstrated in Figure 3.

### 2.4. Postoperative Rehabilitation Protocol

Postoperative rehabilitation was standardized and adjusted according to the presence of concomitant meniscal repair. Continuous passive motion was initiated immediately after surgery to promote early joint mobility. Patients without meniscal repair ambulated with crutches for the first two weeks, progressed to full weightbearing thereafter, and did not require a brace. Jogging was permitted at three months postoperatively, sprinting and competitive exercises at six months, and unrestricted return to sports at approximately nine months. For patients who underwent meniscal repair, a range-of-motion brace was applied for six weeks. Knee flexion was limited to 0–60° during the first four weeks and advanced to full range of motion thereafter. Partial weightbearing with crutches was maintained for four weeks, followed by gradual progression as tolerated.

### 2.5. MRI Evaluation

Postoperative MRI follow-up was performed approximately one year after surgery, depending on patient willingness. The graft was evaluated using proton density sequences.

### 2.6. Statistical Analysis

All statistical analyses were performed using SPSS software (version 23.0; IBM Corp., Armonk, NY, USA) and Microsoft Excel 2016 (Microsoft Corp., Redmond, WA, USA). Categorical variables were analyzed using Fisher’s exact test, and continuous variables were compared using the Mann–Whitney U test or unpaired *t*-test as appropriate. Wilcoxon signed-rank test was used for non-parametric data. Statistical significance was set at a *p*-value less than 0.05.

## 3. Results

A total of 29 patients who underwent combined ACLR and ALLR with suture tape augmentation were identified during the study period. Three patients were excluded due to follow-up of less than one year, and two patients did not complete the PROMs questionnaire. Consequently, 24 patients met the inclusion criteria and were included in the final analysis. The cohort comprised 16 males and 8 females, with a mean age of 30.8 ± 12.8 years (range, 14–50 years). The mean follow-up period was 23.4 ± 6.4 months (range, 12–34 months). Patient demographic and clinical characteristics are summarized in Table 1. All patients presented with a positive pivot-shift test before surgery, including 10 patients with grade 2 and 14 patients with grade 3 instability, indicating significant rotational laxity. Seven patients had ACL graft diameters smaller than 8 mm. Concomitant meniscal injuries were found in 11 patients—eight involving lateral meniscus tears, three with medial meniscus tears and two with concomitant tears. All meniscal tears were treated with arthroscopic repair at the time of reconstruction.

The preoperative and postoperative clinical assessments and patient-reported outcome measures (PROMs) are presented in Table 2. At the final follow-up, patients achieved favorable functional recovery, with mean postoperative IKDC, Marx score and SANE of 83.7 ± 4.8, 9.9 ± 1.6, and 85.2 ± 4.8, respectively. Full range of motion was regained in all patients at an average of 3.2 months postoperatively. Averaged ATT measured by GNRB improved significantly from 4.7 ± 0.7 mm to 1.1 ± 0.4 mm. There were 3 patients who presented with grade 1 pivot shift and 21 with no pivot shift noted post-operatively. Fourteen patients (58.3%) successfully returned to their preinjury activity level within one year after surgery. When patients were stratified by graft diameter (<8 mm vs. ≥8 mm), the baseline demographic characteristics are detailed in Table 3, and the postoperative outcomes and PROMs are summarized in Table 4. Patients in the smaller graft diameter group presented with significantly lower BMI. There were no statistically significant differences between the two groups in postoperative GNRB measurements, pivot-shift grading, or PROMs (all *p* > 0.05).

A single postoperative complication occurred in an 18-year-old patient who developed a knee infection 10 days after reconstruction. The patient presented with knee swelling and erythema and was successfully treated with arthroscopic debridement followed by a two-week course of antibiotics, without recurrence of infection.

Among the 24 patients, 7 patients underwent postoperative magnetic resonance imaging (MRI) one year after surgery. All MRIs demonstrated intact ACL and ALL grafts with normal signal intensity and no significant tunnel widening (>2 mm) was observed in the measured tunnels. Two patients exhibited small cyst-like lesions at the tibial tunnel of ACL, but both were asymptomatic and did not require further intervention. No graft failure or loss of knee stability was observed during the follow-up period.

## 4. Discussion

The results of this study support the hypothesis that combined anterior cruciate ligament reconstruction and anterolateral ligament reconstruction with suture tape augmentation yields promising short-term functional outcomes in patients with ACL injury and pivot-shift instability. The mean postoperative IKDC, Marx activity score, and SANE were 83.7 ± 4.8, 9.9 ± 1.6, and 85.2 ± 4.8, respectively. Full range of motion was restored at an average of 3.2 months postoperatively. There were no significant differences in outcomes between patients with graft diameters < 8 mm and those with grafts ≥ 8 mm. No complications regarding suture tape augmentation, such as foreign body reaction, were observed. Despite the promising outcomes, studies with larger case series, inclusion of control groups, and longer follow-up periods are needed in the future to assess graft protection and durability and validate the reliability of this treatment approach.

Persistent rotational instability after ACL injury has been associated with an increased risk of progressive gonarthrosis, underscoring the essential role of the ACL and rotational knee stability in maintaining joint health and preventing degenerative changes [24]. Combined ACL and ALL reconstruction has gained increasing popularity, particularly in patients at higher risk of residual rotational instability or revision surgeries [25,26]. Recent meta-analyses and randomized controlled trials have reported that combined ACL and ALL reconstruction offers superior control of knee laxity, lower graft failure rates, and higher return-to-sport and graft survival rates [27,28]. The addition of an ALL reconstruction or a lateral extra-articular tenodesis is especially beneficial in high-risk patients, such as young athletes, patients exhibiting high-grade pivot shift, or those with anatomic risks for ACL failure [29,30,31]. All patients in our series presented with significant pivot shift, making them appropriate candidates for combined ACL and ALL reconstruction. After the operation, pivot shift changed from 14 patients with grade III and 10 with grade II preoperatively to 3 with grade I and 21 with grade 0 postoperatively. These findings are in line with previous studies that combined ACLR and ALLR to restore the rotation instability effectively. On the other hand, side to side difference in ATT measured from GNRB reported significant improvement from 4.7 ± 0.7 preoperatively to 1.1 ± 0.4 mm postoperatively. The results were similar to a previous study by Cheng et al., who conducted a study reporting outcome measures of GNRB for ACLR and ALLR [32]. These findings suggested that the augmentation of suture tape in both ACL and ALL constructs may enhance initial stability during the early healing phase. In the present series, no cases of graft failure were observed within the mean follow-up period of two years, consistent with previously reported short-term outcomes.

Suture tape augmentation in ACL reconstruction has been shown in both biomechanical and clinical studies to enhance graft strength, reduce elongation, and provide a “load-sharing” or “safety-belt” effect during early rehabilitation [33,34,35,36,37]. Clinical series have reported low complication rates and satisfactory patient-reported outcomes, with evidence of improved short-term function and decreased re-rupture risk [35,38,39]. Gao et al. reported that ACLR with STA is associated with a reduced graft failure rate and increased RTS rate compared with standard ACLR without additional reoperations or complications [40]. Potential concerns include over-constraint, stress shielding, and delayed graft maturation. Nevertheless, neither animal nor human studies have shown significant differences in ligamentization, histological appearance, or intra-articular complications between augmented and non-augmented grafts [33,41]. Our results were in line with previous studies, with no MRI evidence of tunnel stress shielding or intra-articular abnormal signal change around the ACL graft observed. Regarding the cyst-like lesions observed in two patients on postoperative MRI, these small lesions were located around the tibial tunnel and adjacent to the bioabsorbable screw. They were asymptomatic and were most likely related to the degradation process of the bioabsorbable material rather than the suture tape’s reaction within the bone tunnel. Similar findings have been described in the literature, where bioabsorbable fixation devices were associated with tibial cyst formation after ACL reconstruction [42]. Although these lesions are generally benign and self-limiting, recognizing this imaging appearance is important for accurate interpretation of postoperative MRI findings. Finally, no over-constraint-related range of motion deficits were noted, and all patients regained full range of motion by an average of 3.2 months postoperatively. This might be due to combined STA in ACLR and ALLR allowing early stability and therefore early rehabilitation.

In terms of postoperative rehabilitation, this study adopted the protocol described by Chiu et al., emphasizing early range-of-motion recovery, progressive weight-bearing, and functional training [18]. With recent advances in mechatronic rehabilitation systems integrating robotic assistance and sensor-based feedback, combining such feedback-guided technologies with current rehabilitation strategies could further enhance motion accuracy and neuromuscular re-education [43]. This integration may enable ACL-reconstructed patients to regain natural gait and athletic performance more precisely and efficiently.

Graft diameter is another critical determinant of ACL reconstruction outcomes. Previous studies have demonstrated that hamstring autografts smaller than 8 mm are associated with higher failure and revision rates compared with larger grafts [44,45]. Meta-analyses have shown that each 0.5 mm increase in graft diameter significantly reduces failure risk, and grafts ≥ 8 mm are generally preferred [45,46]. Mariscalco et al. further reported that smaller hamstring autograft size predicted poorer KOOS Sport and Recreation scores at two years postoperatively [47]. On the other hand, a biomechanical study conducted by Bachmaier et al. suggested that STA could reduce the possibility of graft tears, especially for grafts with small diameters [48]. Interestingly, in the present study, no significant difference was found between patients with grafts <8 mm and ≥8 mm in terms of short-term functional outcomes. Both groups demonstrated reliable postoperative stability, with anterior tibial translation (ATT) measuring 1.0 mm and 1.4 mm, respectively. In this study, suture tape augmentation was integrated with both the ACL and ALL grafts, indicating that the reconstructed ACL was reinforced not only by the internal brace within the joint but also by the “ALL–suture tape” construct outside the notch. Therefore, despite smaller graft diameters, satisfactory outcomes could be achieved, as the combined effect of ALL reconstruction and suture tape augmentation likely provided sufficient biomechanical reinforcement to compensate for reduced graft size.

This study has several limitations. First, it was a retrospective study with a relatively small sample size. Second, the mean follow-up period was approximately two years, with a minimum of one year; thus, the results should be considered preliminary. Longer-term follow-up is necessary to evaluate durability, graft maturation, and late complications. Third, there was no control group of isolated ACL reconstruction or ACL plus ALL reconstruction without suture tape augmentation for comparison. Finally, postoperative MRI follow-up was available in only around 30% of patients (7/24), which may have limited the detection of subclinical intra-articular reactions or bone tunnel changes related to the suture tape.

## 5. Conclusions

Combined ACL and ALL reconstruction with suture tape augmentation showed promising short-term results in patients with high-grade pivot shift. The technique was safe, with no range of motion limitation or graft-related complications, and may be a viable option for patients with smaller graft diameters. However, larger prospective comparative studies with extended follow-up are warranted to validate these findings and to further determine the potential advantages of this technique over standard reconstruction methods.

## Figures and Tables

**Table 1:** Patient characteristics.

Age (years)	30.8 ± 12.8
Gender (M/F)	16/8
BMI (kg/m^2^)	20.3 ± 2.0
Side (R/L)	11/13
Graft size (mm)	8.4 ± 0.8
Time from injury (months)	3.9 (2–14) ± 2.3
Follow-up (months)	23.4 (12–34) ± 6.4
Pre-op Pivot grade II/III	10/14
STSD-ATT by GNRB (mm)	4.7 ± 0.7
Medial cartilage injury (n, %)	4 (16.7%)
Lateral cartilage injury (n, %)	3 (12.5%)
Medial meniscus tear (n, %)	3 (12.5%)
Lateral meniscus tear (n, %)	8 (33.3%)

BMI: body mass index; STSD: side-to-side difference; ATT: anterior tibia translation.

**Table 2:** Pre-operative and post-operative PROMs, pivot grade, and ATT.

	Pre-Op (Mean ± SD)	Post-Op (Mean ± SD)	*p*-Value
IKDC	45.9 ± 7.9	83.7 ± 4.8	<0.001
Marx	4.4 ± 1.0	9.9 ± 1.6	<0.001
SANE	49.2 ± 9.7	85.2 ± 4.8	<0.001
Pivot grade (0:1:2:3)	0:0:10:14	21:3:0:0	<0.001
STSD-ATT (mm)	4.7 ± 0.7	1.1 ± 0.4	<0.001

IKDC: International Knee Documentation Committee; SANE: Single Assessment Numeric Evaluation; STSD: side-to-side difference; ATT: anterior tibia translation.

**Table 3:** Pre-operative patient comparison between graft size <8 mm and ≥8 mm.

	<8 mm (n = 7)	≥8 mm (n = 17)	*p*-Value
Age (years)	25.6 ± 14.5	34.4 ± 11.5	0.182
Gender (F/M)	4/3	5/12	0.417
BMI (kg/m^2^)	18.7 ± 1.8	20.9 ± 1.8	0.019
Side (L/R)	3/4	10/7	0.793
Graft size (mm)	7.4 ± 0.2	8.9 ± 0.6	<0.001
Pivot grade (0:1:2:3)	0:0:3:4	0:0:7:10	0.373
STSD-ATT (mm)	4.7 ± 0.8	4.8 ± 0.7	0.782
IKDC	45.4 ± 7.6	46.1 ± 8.2	0.860
Marx	4.9 ± 1.1	4.2 ± 0.9	0.169
SANE	50.7 ± 8.9	48.5 ± 10.3	0.610

BMI: body mass index; STSD: side-to-side difference; ATT: anterior tibia translation; IKDC: International Knee Documentation Committee; SANE: Single Assessment Numeric Evaluation.

**Table 4:** Post-operative PROMs, Pivot test grade, and STSD-ATT by GNRB compared by graft size.

	<8 mm (n = 7)	≥8 mm (n = 17)	*p*-Value
IKDC	82.3 ± 4.2	84.3 ± 5.1	0.334
Marx	9.6 ± 2.2	10.1 ± 1.4	0.605
Sane	86.4 ± 5.6	84.7 ± 4.5	0.485
Pivot grade (0:1:2:3)	6:1:0:0	15:2:0:0	0.881
STSD-ATT (mm)	1.0 ± 0.5	1.1 ± 0.4	0.502

STSD: side-to-side difference; ATT: anterior tibia translation; IKDC: International Knee Documentation Committee; SANE: Single Assessment Numeric Evaluation.

## Data Availability

The datasets generated during or analyzed during the current study are available from the corresponding author on reasonable request.

## Abbreviations

The following abbreviations are used in this manuscript:

ACL	Anterior cruciate ligament
ACLR	Anterior cruciate ligament reconstruction
ALLR	Anterolateral ligament reconstruction
STA	Suture tape augmentation
ATT	Anterior tibial translation
PROMs	Patient-reported outcome measures
IKDC	International Knee Documentation Committee
SANE	Single Assessment Numeric Evaluation
MRI	Magnetic resonance imaging
ROM	Range of motion
BMI	Body mass index
